# Validation of the FVB/N-Tg(HSA* LR)20bCath mice of myotonic dystrophy using swallowing function assessment, histology, and immunofluorescence analysis

**DOI:** 10.1371/journal.pone.0332511

**Published:** 2025-09-12

**Authors:** Rie Asayama, Kaori Tanaka-Nishikubo, Jun Iwanami, Takahiro Fukazawa, Motoi Kanagawa, Naohito Hato

**Affiliations:** 1 Department of Otolaryngology, Head, and Neck Surgery, Ehime University Graduate School of Medicine, Shitsukawa, Toon, Ehime, Japan; 2 Department of Cell Biology and Molecular Medicine, Ehime University Graduate School of Medicine, Shitsukawa, Toon, Ehime, Japan; 3 Division of Medical Research Support, Advanced Research Support Center, Ehime University, Shitsukawa, Toon, Ehime, Japan; University of Minnesota Medical School, UNITED STATES OF AMERICA

## Abstract

Myotonic dystrophy is associated with dysphagia, which can lead to severe complications such as aspiration pneumonia and choking. However, few histopathological studies on dysphagia in myotonic dystrophy have been conducted. In this study, we aimed to validate the FVB/N-Tg(HSA*LR)20bCath mice for studying dysphagia associated with myotonic dystrophy, using videofluoroscopic swallowing study, histological analysis, and immunofluorescence analysis. Videofluoroscopic swallowing study, revealed significant abnormalities during the pharyngeal swallowing phase of swallowing in HSA LR20b mice, including increased pharyngeal residue area and prolonged pharyngeal transit time, suggesting that this mouse model was a valuable tool for studying dysphagia in myotonic dystrophy. These findings might represent a characteristic swallowing pattern in myotonic dystrophy. Histological analysis demonstrated marked variability in muscle fiber size and a high frequency of central nuclei. Additionally, decreased expression of chloride channel 1 was observed in the masseter muscle, suggesting the presence of myotonia. Collectively, these findings provide a foundation for further research into the complex mechanisms underlying myotonic dystrophy associated dysphagia and may inform the development of future treatment strategies.

## Introduction

Myotonic dystrophy (MyD) is an autosomal dominant genetic muscle disorder characterized by progressive muscle weakness, myotonia, and multiple-organ dysfunction [1,2]. MyD is caused by an expansion of Cytosine-Thymine-Guanine (CTG) trinucleotide repeats in the 3′ untranslated region (3′ UTR) of the Dystrophia Myotonica Protein Kinase (*DMPK*) gene located on chromosome 19q13.3 [3,4]. Most of the MyD symptoms result from a toxic gain-of-function mechanism in which mutant *DMPK* RNA accumulates in nuclear ribonuclear foci, sequestering the RNA-binding protein Muscle blind-like protein 1 (MBNL1) and inhibiting normal RNA splicing [5,6], mediated by an expansion of the CTG repeats [7,8]. As the MyD progresses, weakness and myotonia in the distal muscles increase, causing stiffness and debilitating pain, which collectively reduce motor function and quality of life [9]. Although extensive progress has been made in MyD skeletal muscle pathophysiology, the pathophysiology and histopathological characteristics of dysphagia in patients with MyD remain unclear, despite the high incidence of respiratory complications, including aspiration and choking, in MyD patients.

Dysphagia, occurring in 55–80% of patients with MyD [10], is a significant complication that can lead to serious complications such as aspiration pneumonia, malnutrition, and even sudden death due to choking [11–13]. Previous studies have reported that swallowing in patients with MyD is impaired at various stages, from the oral preparatory to the esophageal stage, primarily because of muscle weakness [14–16]. However, patients with MyD often seek medical attention only after their swallowing disorder has become severe and complicated, partly owing to cognitive decline and reluctance to visit hospitals [17,18]. This delay makes pathological diagnosis challenging in many cases. Moreover, owing to ethical limitations in clinical tissue biopsies and the absence of standardized methodologies for animal studies, the precise mechanisms underlying MyD-related dysphagia have remained elusive. Given that dysphagia in patients with MyD is strongly associated with morbidity and mortality, understanding these mechanisms could provide new therapeutic targets that may significantly improve quality of life.

To address these challenges, we utilized Human Skeletal Actin Long Repeat (HSALR) transgenic mice, which are a well-established animal model of MyD [19,20]. These mice are engineered to express an expanded CTG repeat in HSA gene, mimicking the toxic RNA gain-of-function mechanism observed in patients with MyD [21]. HSALR mice exhibit key molecular and clinical features of MyD, including RNA foci accumulation, splicing abnormalities, and myotonia are widely used for animal experiments on MyD [21–24]. Although skeletal muscle pathology has been extensively studied using this model, dysphagia—a clinically significant manifestation of MyD—has not yet been investigated in HSALR mice.

Recently, clinical gold-standard videofluoroscopic swallow study (VFSS) protocols have been adapted for mouse models, offering new opportunities to investigate swallowing pathophysiology [25]. In this study, we performed VFSS in HSALR transgenic mice to characterize swallowing dynamics, evaluating whether they reflect the clinical features of MyD. Furthermore, we combined these assessments with histological and immunofluorescence analyses to explore the potential for therapeutic interventions.

## Materials and methods

### Study animals

Two groups of mice were used in this study, FVB/N-Tg(HSA*LR)20bCath mice (Jackson Laboratory, Stock #032031), hereafter referred to as HSA LR20b mice [21] as the MyD group and FVB/N mice (Japan SLC Co., Ltd.) as control group. The study included 12-month-old mice in both the MyD and control groups. The mice were group-housed based on age and sex (3–6 mice per cage) and kept in a 12 h on and 12 h off lighting cycle in a controlled temperature facility (25 ± 2°C). Food (MF; Oriental Yeast, Tokyo, Japan) and water were provided *ad libitum*, except for overnight prior to VFSS testing. Soiled nesting material was transferred weekly to a new home cage. The results of VFSS and histological and immunofluorescence analyses were compared between the two groups.

All experimental protocols were approved by the Animal Experiment Committee of Ehime University (05HI88−1, 16). Furthermore, all methods were performed in accordance with the relevant ethical guidelines and regulations. This study was conducted in accordance with ARRIVE guidelines.

Animals were assessed at least once daily for general health and behavior, posture, food and water intake. Humane endpoints were defined as follows: body weight loss exceeding 20%, severe respiratory distress, or immobility. If an animal met any of these criteria, it was humanely euthanized using carbon dioxide (CO₂) inhalation, followed by confirmation of death via cervical dislocation. Euthanasia was performed within one hour after endpoint identification.

During the 12-month study period, one mouse was found dead before reaching the humane endpoint criteria. Post-mortem examinations were not conducted; however, based on clinical observations and disease progression, the deaths were presumed to be related to the underlying transgenic phenotype. All efforts were made to minimize suffering. Furthermore, all researchers involved in the study completed institutional training in laboratory animal handling and care, in compliance with the national and international animal welfare standards.

### VFSS equipment and protocol

**Fig 1:**
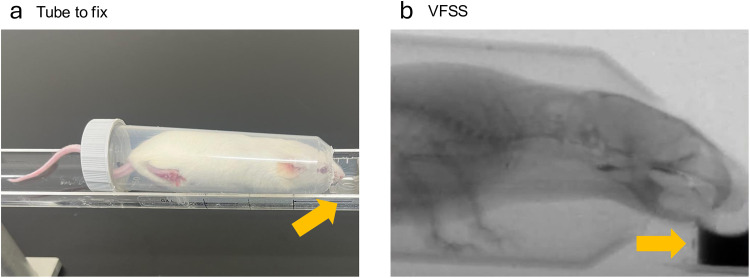
Setting up for VFSS image. **a,** Tube for fixation is adapted using two modifications: one hole is drilled in the cap for the tail, another hole is drilled in the bottom of the tube for the face (arrow: cap of a microtube for contrast agent). **b**, VFSS: swallowing is visualized in the lateral plane. VFSS, videofluoroscopic swallowing study.

The study included 12-month-old mice in both the MyD (n = 9; 6 females, 3 males) and control groups (n = 8; 5 females, 3 males). The VFSS was performed in the lateral plane using the fluoroscopic mode of a micro-computed tomography scanner (CosmoScan GXⅢ; Rigaku, Japan). This device is equipped with the ability to export fluoroscopic movies. During fluoroscopy, the mice were exposed to low-dose radiation (90 kV and 40 μA). Each mouse was placed in a custom-designed conical tube (50 mL) with two modifications: a hole in the cap to allow the mouse’s tail to pass through and another hole at the bottom of the tube to enable the mouse to face outwards. The tube was then fixed to a platform, allowing remote positioning of the mouse within the beam of the fluoroscopy device (Fig 1a). VFSS was performed manually to minimize radiation exposure, with recording initiated when the mouse began drinking.

The contrast agent consisted of iohexol (iodine, 350 mg/mL) diluted 50% in deionized water and chocolate syrup. This liquid was injected into a bowl (substituting the cap of a microtube) using a long tube and syringe, and the mice drank it (Fig 1b).

We conducted a VFSS according to previously published protocol [25] as follows, except that some of the supplies were different. To motivate participation, the mice were subjected to water restriction for 12–16 h prior to the test. To acclimate mice to being in the tube, we started encouraging them to enter the tube 1–2 weeks prior to VFSS testing. In addition, we gave the mice a chocolate-flavored solution in a syringe, without adding contrast agent to assess their reaction to a chocolate syrup.

For each phase of swallowing (from the oropharynx to the esophagus), approximately 10 s of drinking was recorded. VFSS video recordings were captured at 30 frames per second (fps). All video clips for each mouse were later analyzed manually frame-by-frame by two otolaryngologists using video editing software (Dipp-Motion V Ver1.2.5, DITECT, Japan) to obtain the average of three to five consensus values for each swallowing parameter.

Table 1 presents the following measurement endpoints: jaw cycle rate (JCR), jaw excursion distance (JED), inter-swallow interval (ISI), bolus area (BA), pharyngeal residual area (PRA), pharyngeal transit time (PTT), esophageal transit time (ETT). The seven endpoints were evaluated based on previously published methods [25]. In mice, the second cervical vertebra (C2), the most prominent anatomical landmark in the cervical spine, is considered the boundary between the pharynx and the esophagus [25].

**Table 1:** Videofluoroscopic swallowing study parameters.

	Parameters	Explanation
1	Jaw cycle rate	The number of jaw open/close (excursion) cycles during uninterrupted drinking (times/sec).
2	Jaw excursion distance	The distance the jaw opens during jaw excursion cycles, measured between the maxillary and mandibular incisors (mm).
3	Inter-swallow interval (ISI)	The number of video frames between two successive, uninterrupted swallows. The number of video frames is then divided by 30 fps to convert to time (sec).
4	Bolus area (BA)	The BA is measured at the vallecular prior to initiation of the pharyngeal swallow (mm^2^).
5	Pharyngeal residue area (PRA)	The PRA is measured at post-swallow residue in the pharynx (mm^2^).
6	Pharyngeal transit time (PTT)	The PTT is the time required for the bolus through the pharynx. The start frame is ISI start frame, and the end frame is when the tail of the bolus had completely passed the C2. The number of frames divided by 30 fps and converted to time (sec).
7	Esophageal transit time (ETT)	The ETT start frame is identical to the PTT end frame. The ETT end frame is when the bolus has completely entered the stomach. The number of frames is divided by 30 fps and converted to time (sec).

### Histological and immunofluorescence analyses

The animals were anesthetized with medetomidine, midazolam, and butorphanol and euthanized by cervical dislocation. Their tibialis anterior (TA) muscle, sternocleidomastoid muscle (SCM), masseter muscle (MM), digastric muscle (DM), and cricopharyngeal (CP) and thyropharyngeal (TP) muscles were harvested for histological analysis. The mice muscles were frozen in isopentane chilled with liquid nitrogen and subsequently sectioned at 10 μm thickness using a cryostat. Hematoxylin and eosin (H&E) staining was performed according to the standard procedures. H&E staining was performed on muscle samples obtained from the 12-month-old MyD (n = 5; females) and control (n = 5; females) mice. To eliminate potential variability in muscle fiber size due to sex differences, only female mice were used. The analysis focused on muscle fiber size, fiber area variation and the presence of central nuclei in each muscle type. Fiber size is a basic index for evaluating muscle condition. However, since MyD involves both atrophy and hypertrophy of all muscle fibers, it is more accurately evaluated using the variance coefficient (VC) of all muscle fiber for a numerical expression of fiber size variability [26]. The number of fibers with centralized nuclei was counted under field of 10 × objective lens and expressed as a percentage of all fibers. Each muscle images were captured using BZ-X analyzer software (KEYENCE, Japan).

Regarding immunofluorescence analyses, TA muscle, SCM, MM, and DM, those are skeletal muscle, were harvested. Muscle sections were fixed in 4% paraformaldehyde for 15 min, followed by three 5-min washes in phosphate-buffered saline (PBS). Subsequently, the sections were permeabilized for 15 min in 20 mM Tris-HCl (pH 9.0), blocked with 5% bovine serum albumin (BSA)/PBS for 1 h at 4°C, and again washed three times for 5 min in PBS. Sections were immunostained with an antibody against chloride channel-1 (1:50, CLC11-A, Alpha Diagnostic International) targeting chloride channel-1 (ClC-1) at 4°C overnight, which is predominantly expressed in skeletal muscles and plays a crucial role in determining the membrane potential and excitability of skeletal muscle cells. Then, these were immunostained with anti-rabbit Alexa Fluor 488 (1:100, A32790, Thermo Fisher Scientific) as secondary antibody. Myotonia is associated with abnormal alternative splicing of muscle-specific ClC-1 and reduced chloride ion conductance in muscle membranes. Reduced ClC-1 expression is widely recognized as a hallmark indicator of myotonia [27], and has also been observed in patients with MyD. To assess myotonia in individual muscles, immunostaining with an antibody against ClC-1 was performed on samples from the MyD (n = 8) and control (n = 7) mice. Considering quantitation of ClC-1 immunoreactivity, three separate regions of images under 10 × objective lens were randomly selected and automatically measured using BZ-X analyzer software. Microscope exposure was controlled at a fixed level.

### Statistical analyses

All statistical analyses were performed using EZR (Saitama Medical Center, Jichi Medical University, Saitama, Japan), which is a graphical user interface for R (R Foundation for Statistical Computing, Vienna, Austria) software. It is a modified version of R commander software designed to add statistical functions frequently used for biostatistics [28]. Comparisons of metrics between the two groups were performed using Mann–Whitney U or Fisher’s exact tests. Statistical significance was set at p < 0.05 for all tests.

## Results

### Swallowing movement force is decreased in HSA LR20b mice

**Fig 2:**
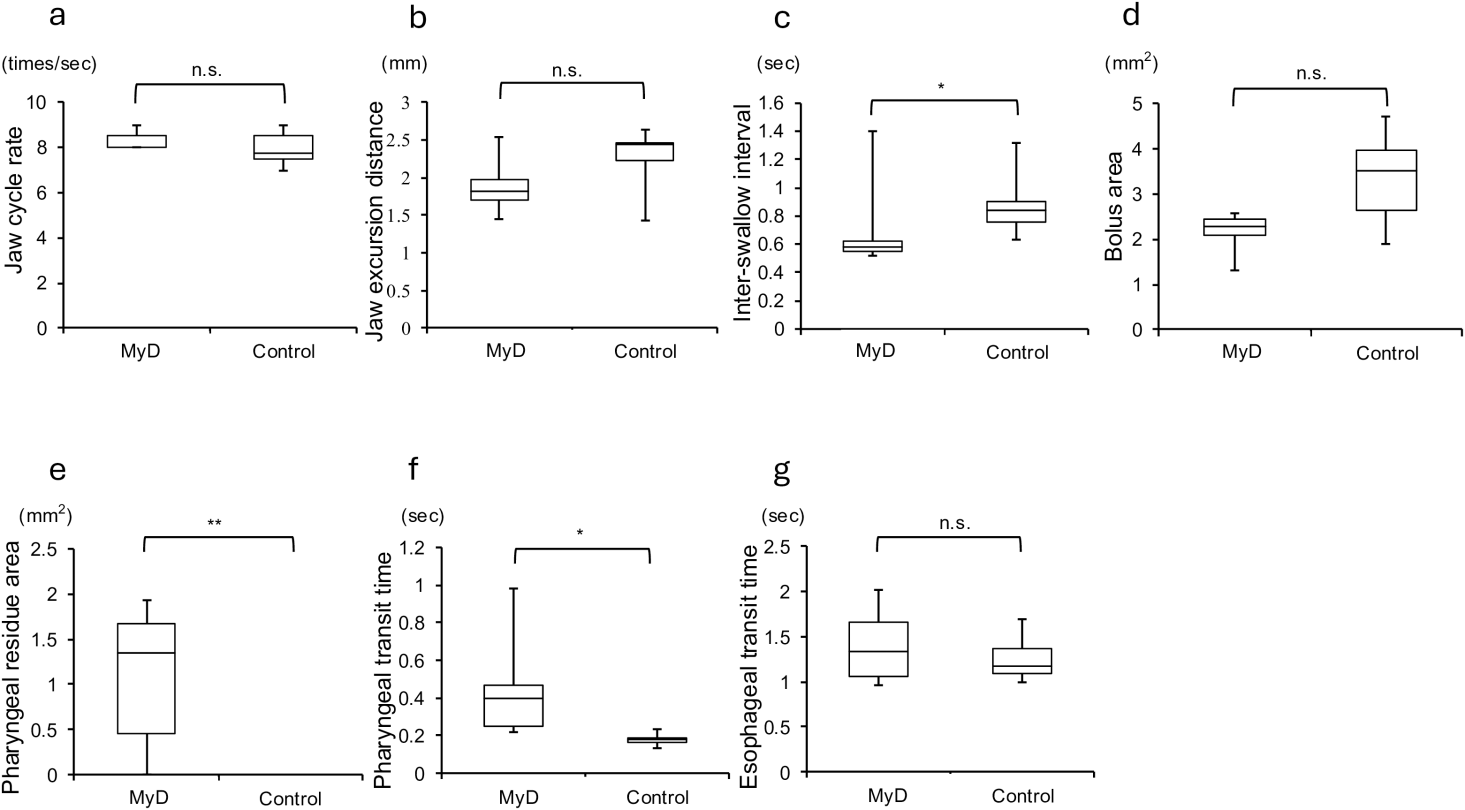
VFSS results. **a**, jaw cycle rate; **b**, jaw excursion distance; **c**, Inter-swallow interval; **d**, bolus area; **e**, pharyngeal residue area; **f**, pharyngeal transit time; **g**, esophageal transit time. There was significant increases in the PRA (p = 0.001) and duration of the PTT (p = 0.001); the ISI was significantly shorter (p = 0.01) in the MyD group. ISI, Inter-swallow interval; MyD, myotonic dystrophy; PRA, pharyngeal residual area; PTT, pharyngeal transit time; VFSS, videofluoroscopic swallowing study.

In the MyD group, significant increases in PRA (p = 0.001) and duration of PTT (p = 0.001) were observed (Fig 2e, 2f).

No significant differences were observed in JCR, JED, BA, and ETT within the MyD group (Fig 2a, 2b, 2d, 2g). However, the ISI was significantly shorter (p = 0.01, Fig 2c).

### Dystrophic changes in swallowing-related muscles of HSA LR20b mice observed by H&E staining

**Fig 3:**
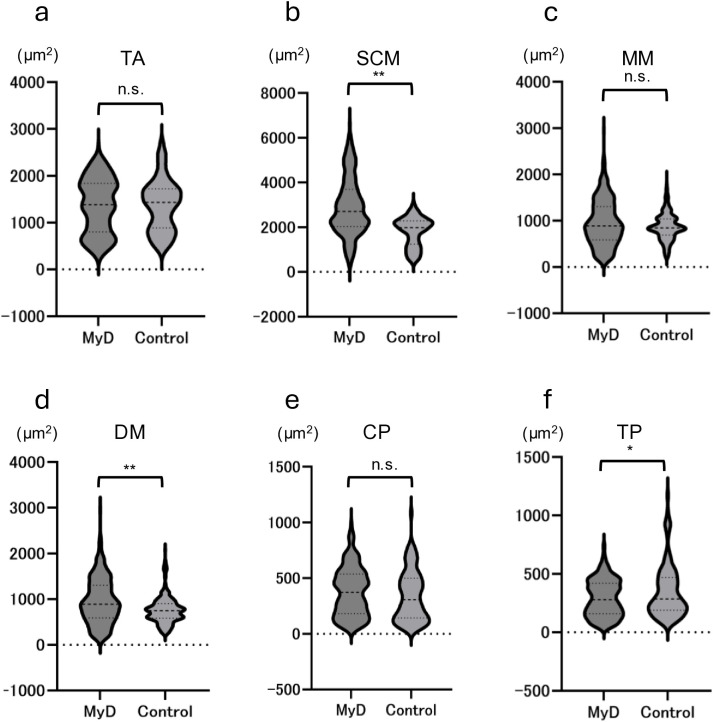
Fiber size and the VC. **a**, Tibialis anterior muscle; **b**, sternocleidomastoid muscle; **c**, masseter muscle; **d**, digastric muscle; The MyD group exhibits increased fiber area variation in the tibialis anterior, sternocleidomastoid, masseter, and digastric muscles. The vertical spread shows the size of the VC. VC, variance coefficient; MyD, myotonic dystrophy.

Regarding fiber size, SCM and DM were larger in the MyD group. TP was larger in the control group. VC was elevated in the TA, SCM, MM, and DM compared with the control group (Fig 3). Although VC was higher in the CP compared with other muscles, no difference was much observed between the MyD and control groups.

**Fig 4:**
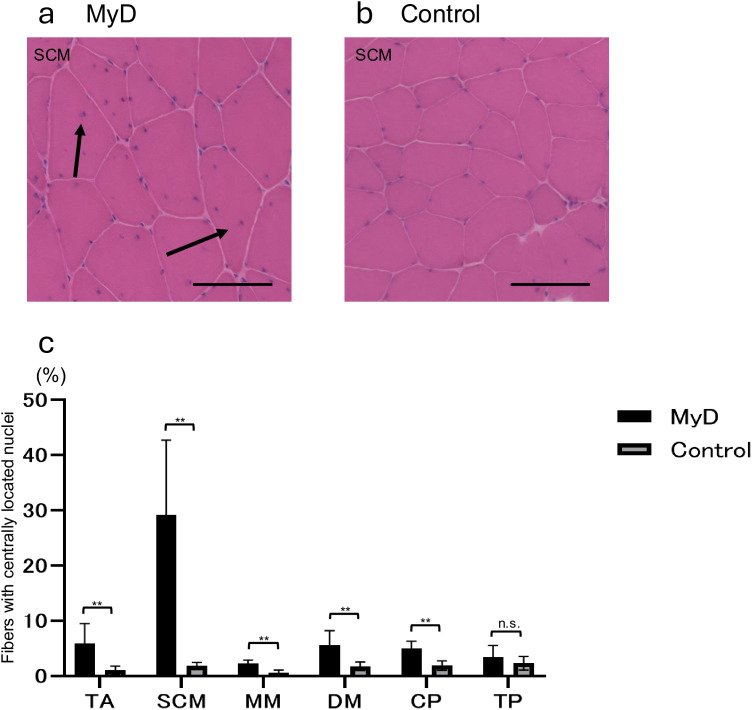
H&E staining of the sternocleidomastoid muscle. Arrow shows central nuclei; scale bar, 100 μm; **a**, MyD; **b**, control; **c**. central nuclei, number of central nuclei/cell count ×100 (%). Central nuclei were observed significantly more frequently in the tibialis anterior, sternocleidomastoid, masseter, digastric, and cricopharyngeal muscles of the MyD mice, whereas they are rarely observed in control mice. H&E, hematoxylin and eosin; MyD, myotonic dystrophy.

In contrast, central nuclei, a marker of MyD, were significantly more frequently observed in approximately all examined muscles of MyD mice, including the TA, SCM, MM, DM, and CP (Fig 4).

### Assessment of myotonia in the masseter muscle of HSA LR20b mice by immunofluorescence

**Fig 5:**
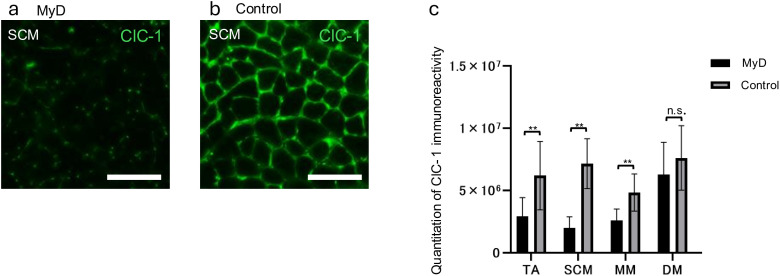
Expression of ClC-1. scale bar, 100 μm; **a**, MyD (sternocleidomastoid muscle); **b**, control (sternocleidomastoid muscle); **c**, each muscle; ClC-1 expression is significantly decreased in the tibialis anterior, sternocleidomastoid, and masseter muscles in the MyD group compared with those in the control group. ClC-1, chloride channel 1; MyD, myotonic dystrophy.

Myotonia was further assessed by evaluating ClC-1 expression, which was significantly reduced in the TA, SCM, and MM muscles in the MyD group compared with controls, suggesting the involvement of myotonia (Fig 5).

## Discussion

In this study, we conducted the first evaluation of swallowing dynamics using VFSS and histological and immunofluorescence analysis of swallowing-related muscles in HSA LR20b mice, a model of MyD. The present findings suggest that HSA LR20b mice exhibited swallowing dysfunctions resembling those observed in clinical settings [29], characterized by increased PRA and prolonged PTT, supporting their potential utility as a dysphagia model relevant to MyD. Additionally, histological examination and immunofluorescence staining revealed that dystrophic changes were generally evident in muscles associated with swallowing, with myotonia specifically observed in the masseter muscle. These results indicated potential areas of interest for future studies aimed to develop treatments of MyD.

VFSS examination of HSA LR20b mice revealed characteristic swallowing dysfunctions, specifically increased PRA and a prolonged duration of PTT. These findings likely reflect impaired pharyngeal-to-esophageal transit, suggesting a reduction in pharyngeal contractile force. Furthermore, the observed shortening of the ISI may represent a compensatory mechanism to overcome decreased pharyngeal propulsion. These patterns closely mirror the dysphagic features commonly observed in patients with MyD, such as pharyngeal residue or reduction in contraction pressure in the pharynx [2,16], thereby supporting the clinical relevance of the HSA LR20b mouse model for studying swallowing dysfunction.

In previous reviews, videofluorography is acknowledged as the gold standard for functional assessment of swallowing in animal dysphagia studies. To date, VFSS has been reported in species such as dogs, rats, and mice [30–32]. Regarding dog VFSS, structures such as the epiglottis, soft palate, and nasopharynx can be clearly visualized; laryngeal excursion can also be evaluated. This enables more comprehensive assessment of swallowing dynamics and making this modality useful for studying dysphagia pathophysiology [32]. Larger animals generally exhibit lower X-ray permeability, which enhances the visibility of swallowing-related structures. However, currently, the animal model of MyD targeted in this study has only been established in mice. Due to the higher X-ray permeability in mice, there are limitations in visualizing certain swallowing-related structures under fluoroscopy.

Nonetheless, Russell et al. reported that “rats with nigrostriatal dopamine depletion exhibit significantly prolonged PTT, indicating neuromuscular discoordination” [31]. Lever et al, a study using mice, noted that “aging mouse were observable as increases in BA and prolonged PTT and ETT in VFSS metrics”, which closely resembled the clinical signs of neuromuscular deficits [33]. Taken together, prolonged PTT observed in the current model are considered to reflect dysphagia underlying neuromuscular impairments.

Although HSA LR20b mice exhibit swallowing dysfunctions similar to those seen in patients with MyD, species-specific anatomical and physiological differences between mice and humans may influence the clinical applicability of these findings. These differences underscore the need for caution when interpreting murine VFSS data in translational contexts.

With H&E staining, central nuclei were observed in all muscles except the TP; dystrophic changes were generally evident in muscles associated with swallowing in 12-month-old MyD mice. This finding suggests a decline in pharyngeal-to-esophageal propulsive force, as reflected by increased PRA and prolonged PTT using VFSS.

Furthermore, immunofluorescence revealed that ClC-1 expression was significantly reduced in the MM of the MyD model, suggesting the presence of myotonia in the MM. Myotonia is a state characterized by delayed muscle relaxation following contraction due to increased excitability of skeletal muscles; it is a hallmark feature of MyD [34].

Among the VFSS parameters related to the oral preparatory phase, jaw cycle rate and jaw excursion distance were assessed as indicators of masticatory function. Although no significant differences were found, the jaw excursion distance tended to be reduced in the MyD group. In MyD cases, choking partly caused by chewing disorders is often a leading cause of death. Swallowing dysfunction in patients with MyD, often goes unrecognized and untreated due to factors such as cognitive impairment, lack of disease insight, and resistance to seeking medical care. As a result, aspiration pneumonia and choking remain significant causes of mortality in this population.

Since the MM plays an essential role in chewing, its dysfunction may contribute to impaired mastication of solid food, which is strongly associated with an increased risk of choking. Myotonia is linked to aberrant alternative splicing of the muscle-specific CLCN1 gene, resulting in reduced chloride ion conductance across muscle membranes. Consequently, reduced ClC-1 expression is widely accepted as a biomarker of myotonia. Several therapeutic interventions—such as antisense oligonucleotides [35], calcitriol [36], pentamidine [37], and erythromycin [38]—have been reported to alleviate myotonia in animal models. These findings suggest a potential therapeutic avenue for addressing masticatory dysfunction in MyD cases, warranting further preclinical and clinical investigation. However, recent studies have proposed that a combination of ClC-1 dysfunction and calcium channel abnormalities may synergistically contribute to muscle pathology in patients with MyD [39]. A comprehensive understanding of these mechanisms will require further investigation into other ion transport molecules, including calcium and sodium channels. Furthermore, the absence of ClC-1 expression has also been associated with increased susceptibility to aging [40]. This discrepancy may be due to differences in animal species, assessment methodologies, or technical limitations, warranting further investigation.

In the present study, we confirmed dystrophic changes across approximately all swallowing-related muscles, as well as the presence of myotonia specifically in the MM. These findings highlight the critical need for early intervention in patients with MyD to address swallowing and masticatory dysfunction. Adjusting food textures and implementing targeted therapeutic interventions based on functional assessment could potentially reduce the incidence of choking and aspiration pneumonia, thereby improving patient outcomes. Future studies are warranted to further explore and validate effective intervention strategies in this vulnerable population.

### Limitations

This study had some limitations that should be considered. First, since we focused exclusively on 12-month-old mice, our understanding of the progression of dysphagia across different age groups remains limited. Future studies should include mice of varying ages to gain a more comprehensive understanding of how impairment progresses over time. Second, there were some concerns about VFSS methodology. Although we employed VFSS to assess swallowing dynamics, the soft tissue structures involved in the mouse swallowing mechanism (e.g., the tongue, velum, posterior pharyngeal wall, and epiglottis) were not clearly visible using current fluoroscopy systems. This limitation hindered our ability to directly evaluate the biomechanics of swallowing. Additionally, VFSS video recording was restricted to 30 fps, which was insufficient to capture all phases of rapid swallowing movements in mice, which occur at higher frequency than in humans. Future studies would benefit from using advanced imaging technologies, such as high-frame-rate cameras, to overcome this limitation. Furthermore, in this study, we did the conventional VFSS method more concisely; we did not assess whether the mice were stressed by being fixed in a conical tube and whether it affected the results. We need to consider whether it would be possible to conduct video recording with a larger size container in the future. Finally, this study focused solely on ClC-1 as a marker of MyD; other ion channels and molecular mechanisms should also be investigated to provide a deeper understanding of this process. Addressing these limitations in future studies will be essential for advancing our understanding of dysphagia in myotonic dystrophies and developing effective treatment strategies.

## Conclusions

Our findings using a modified VFSS protocol demonstrated that the HSA LR20b mouse model effectively replicated key features of dysphagia observed in patients with MyD, establishing it as a valuable tool for studying disease-associated swallowing dysfunction. Furthermore, decreased ClC-1 expression in the MM indicated the presence of myotonia, highlighting the potential for future research into therapeutic interventions targeting impaired masticatory function in patients with MyD. This study provides important insights into the mechanisms underlying MyD-associated dysphagia and emphasizes the critical need for continued research to improve diagnostic accuracy and to develop more effective treatment strategies for managing dysphagia in patients with MyD.

## References

[1] Wheeler TM, Thornton CA. Myotonic dystrophy: RNA-mediated muscle disease. Curr Opin Neurol. 2007;20(5):572–6. doi: 10.1097/WCO.0b013e3282ef6064 17885447

[2] Pilz W, Baijens LWJ, Kremer B. Oropharyngeal dysphagia in myotonic dystrophy type 1: a systematic review. Dysphagia. 2014;29(3):319–31. doi: 10.1007/s00455-013-9510-9 24458731

[3] Mahadevan M, Tsilfidis C, Sabourin L, Shutler G, Amemiya C, Jansen G, et al. Myotonic dystrophy mutation: an unstable CTG repeat in the 3’ untranslated region of the gene. Science. 1992;255(5049):1253–5. doi: 10.1126/science.1546325 1546325

[4] Bird TD. Myotonic dystrophy type 1. In: Adam MP ed. GeneReviews®. Seattle, WA. 1993.

[5] Miller JW, Urbinati CR, Teng-Umnuay P, Stenberg MG, Byrne BJ, Thornton CA, et al. Recruitment of human muscleblind proteins to (CUG)(n) expansions associated with myotonic dystrophy. EMBO J. 2000;19(17):4439–48. doi: 10.1093/emboj/19.17.4439 10970838 PMC302046

[6] Mahadevan MS, Yadava RS, Mandal M. Cardiac pathology in myotonic dystrophy Type 1. Int J Mol Sci. 2021;22(21):11874. doi: 10.3390/ijms222111874 34769305 PMC8584352

[7] Lee JE, Cooper TA. Pathogenic mechanisms of myotonic dystrophy. Biochem Soc Trans. 2009;37(Pt 6):1281–6. doi: 10.1042/BST0371281 19909263 PMC3873089

[8] Chau A, Kalsotra A. Developmental insights into the pathology of and therapeutic strategies for DM1: back to the basics. Dev Dyn. 2015;244(3):377–90. doi: 10.1002/dvdy.24240 25504326

[9] Galli M, Cimolin V, Crugnola V, Priano L, Menegoni F, Trotti C, et al. Gait pattern in myotonic dystrophy (Steinert disease): a kinematic, kinetic and EMG evaluation using 3D gait analysis. J Neurol Sci. 2012;314(1–2):83–7. doi: 10.1016/j.jns.2011.10.026 22118863

[10] Hilbert JE, Ashizawa T, Day JW, Luebbe EA, Martens WB, McDermott MP, et al. Diagnostic odyssey of patients with myotonic dystrophy. J Neurol. 2013;260(10):2497–504. doi: 10.1007/s00415-013-6993-0 23807151 PMC4162528

[11] LaDonna KA, Koopman WJ, Venance SL. Myotonic dystrophy (DM1) and dysphagia: the need for dysphagia management guidelines and an assessment tool. Can J Neurosci Nurs. 2011;33(1):42–6. 21560885

[12] Mathieu J, Allard P, Potvin L, Prévost C, Bégin P. A 10-year study of mortality in a cohort of patients with myotonic dystrophy. Neurology. 1999;52(8):1658–62. doi: 10.1212/wnl.52.8.1658 10331695

[13] de Die-Smulders CE, Höweler CJ, Thijs C, Mirandolle JF, Anten HB, Smeets HJ, et al. Age and causes of death in adult-onset myotonic dystrophy. Brain. 1998;121 ( Pt 8):1557–63. doi: 10.1093/brain/121.8.1557 9712016

[14] Swick HM, Werlin SL, Dodds WJ, Hogan WJ. Pharyngoesophageal motor function in patients with myotonic dystrophy. Ann Neurol. 1981;10(5):454–7. doi: 10.1002/ana.410100508 7305298

[15] Eckardt VF, Nix W, Kraus W, Bohl J. Esophageal motor function in patients with muscular dystrophy. Gastroenterology. 1986;90(3):628–35. doi: 10.1016/0016-5085(86)91117-0 3943694

[16] Costantini M, Zaninotto G, Anselmino M, Marcon M, Iurilli V, Boccù C, et al. Esophageal motor function in patients with myotonic dystrophy. Dig Dis Sci. 1996;41(10):2032–8. doi: 10.1007/BF02093607 8888718

[17] Harvey JC, Sherbourne DH, Siegel CI. Smooth muscle involvement in myotonic dystrophy. Am J Med. 1965;39:81–90. doi: 10.1016/0002-9343(65)90247-0 14314240

[18] Bungener C, Jouvent R, Delaporte C. Psychopathological and emotional deficits in myotonic dystrophy. J Neurol Neurosurg Psychiatry. 1998;65(3):353–6. doi: 10.1136/jnnp.65.3.353 9728948 PMC2170247

[19] Carrascosa-Sàez M, Colom-Rodrigo A, González-Martínez I, Pérez-Gómez R, García-Rey A, Piqueras-Losilla D, et al. Use of HSALR female mice as a model for the study of myotonic dystrophy type I. Lab Anim (NY). 2025;54(4):92–102. doi: 10.1038/s41684-025-01506-7 40016516 PMC11957995

[20] Kanadia RN, Shin J, Yuan Y, Beattie SG, Wheeler TM, Thornton CA, et al. Reversal of RNA missplicing and myotonia after muscleblind overexpression in a mouse poly(CUG) model for myotonic dystrophy. Proc Natl Acad Sci U S A. 2006;103(31):11748–53. doi: 10.1073/pnas.0604970103 16864772 PMC1544241

[21] Mankodi A, Logigian E, Callahan L, McClain C, White R, Henderson D, et al. Myotonic dystrophy in transgenic mice expressing an expanded CUG repeat. Science. 2000;289(5485):1769–73. doi: 10.1126/science.289.5485.1769 10976074

[22] Falcetta D, Quirim S, Cocchiararo I, Chabry F, Théodore M, Stiefvater A, et al. CaMKIIβ deregulation contributes to neuromuscular junction destabilization in myotonic dystrophy type I. Skelet Muscle. 2024;14(1):11. doi: 10.1186/s13395-024-00345-3 38769542 PMC11106974

[23] Chen G, Masuda A, Konishi H, Ohkawara B, Ito M, Kinoshita M, et al. Phenylbutazone induces expression of MBNL1 and suppresses formation of MBNL1-CUG RNA foci in a mouse model of myotonic dystrophy. Sci Rep. 2016;6:25317. doi: 10.1038/srep25317 27126921 PMC4850456

[24] Mankodi A, Takahashi MP, Jiang H, Beck CL, Bowers WJ, Moxley RT, et al. Expanded CUG repeats trigger aberrant splicing of ClC-1 chloride channel pre-mRNA and hyperexcitability of skeletal muscle in myotonic dystrophy. Mol Cell. 2002;10(1):35–44. doi: 10.1016/s1097-2765(02)00563-4 12150905

[25] Lever TE, Braun SM, Brooks RT, Harris RA, Littrell LL, Neff RM, et al. Adapting human videofluoroscopic swallow study methods to detect and characterize dysphagia in murine disease models. J Vis Exp. 2015;(97):52319. doi: 10.3791/52319 25866882 PMC4401177

[26] Rüegg MA, Meinen A. Quantitative determination of minimal Feret’s diameter, including the evaluation of the percentage of centralized nuclei, fiber numbers, cross-sectional area and the percentage of fibrosis. Biozentrum, University of Basel; 2011.

[27] Hu N, Kim E, Antoury L, Wheeler TM. Correction of Clcn1 alternative splicing reverses muscle fiber type transition in mice with myotonic dystrophy. Nat Commun. 2023;14(1):1956. doi: 10.1038/s41467-023-37619-1 37029100 PMC10082032

[28] Kanda Y. Investigation of the freely available easy-to-use software “EZR” for medical statistics. Bone Marrow Transp. 2013;48(3):452–8. doi: 10.1038/bmt.2012.244 23208313 PMC3590441

[29] Waito AA, Valenzano TJ, Peladeau-Pigeon M, Steele CM. Erratum to: trends in research literature describing dysphagia in motor neuron diseases (MND): a scoping review. Dysphagia. 2017;32(6):748. doi: 10.1007/s00455-017-9829-8 28664472 PMC5724560

[30] Kim H-N, Kim J-Y. a systematic review of oropharyngeal dysphagia models in rodents. Int J Environ Res Public Health. 2021;18(9):4987. doi: 10.3390/ijerph18094987 34067192 PMC8125817

[31] Russell JA, Ciucci MR, Hammer MJ, Connor NP. Videofluorographic assessment of deglutitive behaviors in a rat model of aging and Parkinson disease. Dysphagia. 2013;28(1):95–104. doi: 10.1007/s00455-012-9417-x 22763806 PMC3554861

[32] Harris RA, Grobman ME, Allen MJ, Schachtel J, Rawson NE, Bennett B, et al. Standardization of a videofluoroscopic swallow study protocol to investigate dysphagia in dogs. J Vet Intern Med. 2017;31(2):383–93. doi: 10.1111/jvim.14676 28240398 PMC5354069

[33] Lever TE, Brooks RT, Thombs LA, Littrell LL, Harris RA, Allen MJ, et al. Videofluoroscopic validation of a translational murine model of presbyphagia. Dysphagia. 2015;30(3):328–42. doi: 10.1007/s00455-015-9604-7 25783697

[34] Wheeler TM, Lueck JD, Swanson MS, Dirksen RT, Thornton CA. Correction of ClC-1 splicing eliminates chloride channelopathy and myotonia in mouse models of myotonic dystrophy. J Clin Invest. 2007;117(12):3952–7. doi: 10.1172/JCI33355 18008009 PMC2075481

[35] Stoodley J, Vallejo-Bedia F, Seone-Miraz D, Debasa-Mouce M, Wood MJA, Varela MA. Application of antisense conjugates for the treatment of myotonic dystrophy Type 1. Int J Mol Sci. 2023;24(3):2697. doi: 10.3390/ijms24032697 36769018 PMC9916419

[36] Huang K, Wang D-D, Hu W-B, Zeng W-Q, Xu X, Li Q-X, et al. Calcitriol increases MBNL1 expression and alleviates myotonic dystrophy phenotypes in HSALR mouse models. J Transl Med. 2022;20(1):588. doi: 10.1186/s12967-022-03806-9 36510245 PMC9743610

[37] Coonrod LA, Nakamori M, Wang W, Carrell S, Hilton CL, Bodner MJ, et al. Reducing levels of toxic RNA with small molecules. ACS Chem Biol. 2013;8(11):2528–37. doi: 10.1021/cb400431f 24028068 PMC4108295

[38] Nakamori M, Taylor K, Mochizuki H, Sobczak K, Takahashi MP. Oral administration of erythromycin decreases RNA toxicity in myotonic dystrophy. Ann Clin Transl Neurol. 2015;3(1):42–54. doi: 10.1002/acn3.271 26783549 PMC4704483

[39] Nitschke L, Cooper TA. Combinatorial effects of ion channel mis-splicing as a cause of myopathy in myotonic dystrophy. J Clin Invest. 2024;134(1):e176089. doi: 10.1172/JCI176089 38165037 PMC10760967

[40] Conte E, Fonzino A, Cibelli A, De Benedictis V, Imbrici P, Nicchia GP, et al. Changes in expression and cellular localization of rat skeletal muscle ClC-1 chloride channel in relation to age, myofiber phenotype and PKC modulation. Front Pharmacol. 2020;11:714. doi: 10.3389/fphar.2020.00714 32499703 PMC7243361

